# Physical activity and lifestyle management after joint replacement surgery (PALMS): A feasibility study

**DOI:** 10.1016/j.ocarto.2026.100815

**Published:** 2026-05-12

**Authors:** Christine Bowman, Casey L. Peiris, Lyndon J. Hawke, Shae Cooke, Claire Wundersitz, Cathy Senserrick, Alice Embry, Linda Wubbeling, Nicholas F. Taylor

**Affiliations:** a Community Rehabilitation Program, Eastern Health, Australia; b Department of Physiotherapy, Eastern Health, Australia; c Allied Health Clinical Research Office, Eastern Health, Australia; d Allied Health, Royal Melbourne Hospital, Australia; e School of Allied Health, Human Services and Sport, La Trobe University, Australia

**Keywords:** Exercise-based rehabilitation, TKR, THR, Exercise therapy, Feasibility

## Abstract

**Objective:**

To assess the feasibility of a program to increase physical activity as part of lifestyle management after joint replacement surgery (PALMS).

**Design:**

This pragmatic pre-post feasibility study included participants referred to community rehabilitation post total hip or knee replacement surgery. Participants attended in-person for 1 h, twice-weekly for six weeks. PALMS consisted of a health screen, including point-of-care testing for metabolic syndrome, a group exercise program based on a cardiac rehabilitation model, and education. Feasibility outcomes were acceptability, demand, implementation and practicality. Secondary exploratory outcomes included physical activity and physical functioning.

**Results:**

Forty-six participants (23 men, 23 women), mean age 62 (SD 8) years and a mean of 7 (SD 4) weeks post knee (n = 27) or hip (n = 19) joint replacement surgery were enrolled. PALMS was well accepted by participants and clinicians, was implemented with high rates of adherence (mean 10.4/12 (SD 3.0) sessions attended), and 76% (28 of 37) had metabolic syndrome indicating high demand. However, recruitment did not meet threshold targets of 7 recruits each month (achieved 5.1) and 50% of eligible clients agreeing to participate (achieved 38%). There were no serious adverse events. Post-program, physical activity (mean change 1,678 METS min/wk, 95%CI 997 to 2,359) and physical functioning (6-min walk test mean change 167 m, 95%CI 120 to 215) increased.

**Conclusion:**

The PALMS group program was feasible and associated with short-term improvements in physical activity and physical functioning. With attention paid to recruitment strategies the next step is to evaluate program effectiveness with an adequately powered trial.

## 1 Introduction

Individuals with osteoarthritis who require lower-limb joint replacement have almost twice the risk of dying from cardiovascular disease compared to the general population, with physical activity levels significantly lower than age-matched peers [1]. According to physical activity guidelines, adults should complete a minimum of 150 min of moderate intensity cardiovascular exercise or 75 min of vigorous cardiovascular exercise per week to achieve health benefits [2]. Adequate levels of physical activity are associated with positive health outcomes including reduced cardiovascular risk. After lower limb joint replacement surgery there is often relief of osteoarthritic symptoms, but physical activity levels do not increase [3,4]. Therefore, these people likely remain at increased risk of cardiovascular disease.

Current models of rehabilitation after joint replacement focus on reducing impairment at the affected joint, for example increasing muscle strength [5] and increasing functional activity associated with the affected joint (e.g. control of sit to stand) [6]. Clinical practice guidelines for total knee joint replacement recommend physiotherapists teach patients the importance of progression of physical activity [6]. However, the proposed benefits of increased physical activity in these guidelines were improved gait function and activity, rather than broader health benefits. Consequently, current models of rehabilitation after joint replacement do not address the health of the whole person by addressing the increased risk of cardiovascular disease that is typical of this population.

Metabolic syndrome is a set of signs that indicate an increased risk of developing chronic diseases such as cardiovascular disease and diabetes [7]. Metabolic syndrome is also part of the aetiology of osteoarthritis due to high levels of chronic systemic inflammation present in both conditions [8,9]. To test positive for metabolic syndrome, an individual must have three of five metabolic risk factors: increased waist circumference, increased blood pressure, increased levels of blood glucose, low levels of HDL-cholesterol and raised triglycerides [10]. About 25% of all adults have metabolic syndrome, but prevalence is doubled to about 50% of those with osteoarthritis [11,12]. Another study found metabolic syndrome in adults with osteoarthritis was associated with poorer clinical outcomes [13]. In addition, 64% of people presenting to a community rehabilitation program for allied health management (primarily for musculoskeletal complaints) had metabolic syndrome, yet it had only previously been diagnosed in 2% of them [14]. These findings suggest that patients post joint replacement may be a group with substantial, often unrecognised cardiometabolic risk, as indicated by high rates of metabolic syndrome, highlighting the potential value of a more holistic approach to rehabilitation.

Lifestyle management comprising exercise and diet is recommended first-line management for people with metabolic syndrome [7] and people with osteoarthritis [15,16]. Lifestyle management includes interventions that aim to increase physical activity, modify diet and manage weight for non-communicable diseases such as osteoarthritis, cardiovascular diseases and metabolic syndrome by encouraging long-term behaviour change [17]. Participation in these types of interventions can reverse metabolic syndrome in up to 40% of participants [18,19]. Cardiac rehabilitation, typically comprising exercise and education, has been associated with a 32% reduction in mortality for those with cardiovascular disease [20]. Therefore, lifestyle management is appropriate and would potentially be beneficial post joint replacement surgery. A key component of lifestyle management is behaviour change [21]. The observed lack of change in physical activity after total joint replacement [3,4] may be because rehabilitation programs often lack structured behavioral strategies designed to facilitate or support changes in physical activity.

In summary, current models of rehabilitation after total joint replacement that focus on reducing joint impairment and activity limitation may not improve the general health of participants. A new holistic approach based on the cardiac rehabilitation model incorporating elements of behaviour change may be required. However, there is no evidence that such a model will be feasible and effective in this population. Therefore, the first step in evaluating a new model of rehabilitation after total joint replacement is to evaluate feasibility. Our primary aim was to assess the feasibility of delivering a new model of rehabilitation designed to increase physical activity as part of lifestyle management post joint replacement surgery. Bowen's framework guided the assessment of feasibility under the following domains: acceptability, demand, implementation and practicality [22]. Our secondary aims were to estimate the effect of the new model of rehabilitation on increasing physical activity, improving diet, reducing metabolic risk factors, increasing knowledge of physical activity guidelines, and improving self-reported function at the hip and knee.

## 2 Methods

### 2.1 Study design and participants

A pragmatic, single group, pre-post study with measures taken at baseline (T0) and after a six-week (T1) rehabilitation group intervention called PALMS (Physical Activity and Lifestyle Management after joint replacement Surgery) was completed at a publicly funded community rehabilitation program in metropolitan Melbourne, Australia. Participants were recruited from March 5, 2024 to January 2, 2025 with the last assessment completed on April 14, 2025. Community rehabilitation programs offer centre-based and home-based allied health rehabilitation delivered individually or in groups for community-dwelling individuals requiring multidisciplinary management. The study received ethics committee approval (LR24-007-105062) and all participants completed written informed consent. The study is reported consistent with strengthening the reporting of observational studies in epidemiology [23].

There were some minor variations to the study protocol approved by the ethics committee. First, it was intended to complete point-of-care testing on all participants for whom triglycerides and HDL cholesterol (HDL-C) risk factor status was unknown (i.e. they did not have documented blood test results and were not on medications). However, the testing kit for testing triglycerides and HDL-C was not functioning consistently; therefore, there were missing baseline data for these metabolic risk factors. Second, to ease participant burden the dietary assessment was not repeated at T1 as planned. However, T0 measures were retained to describe participant’s typical diet to determine whether there was a need for dietary intervention.

Participants were eligible if referred to the community rehabilitation program post elective total hip or knee replacement surgery. Those who were unable to participate in a group setting were not eligible, for example if they had cognitive limitations, post-surgical complications, unsafe mobility, or if they were unable to access the centre-based program. Medical clearance was obtained prior to participation in cases where exercise may have been contraindicated due to medical instability.

### 2.2 Procedures and interventions

All participants received standard physiotherapy care within the community rehabilitation program comprising an initial 60-min assessment plus a further one to two physiotherapy 60-min appointments until mobility and pain control was judged sufficient to commence PALMS. Once safe to attend the group, T0 assessments were completed and participants attended the PALMS group twice-weekly for 1 h for six weeks (12 sessions in total). PALMS consisted of a health screen - including point of care testing for metabolic syndrome and questionnaires to assess lifestyle risk factors - followed by the group exercise program and education (Table 1) [24]. There were a maximum of 8 participants in each group, which was led by a physiotherapist assisted by either a physiotherapist or an allied health assistant.

**Table 1:** Description of study according to the template for intervention description and replication (TIDieR) [22].

	Intervention
Brief name	Physical activity and lifestyle management after joint replacement surgery (PALMS)
Why	To promote health and reduce cardiovascular risk factors post total joint replacement
What materials	CardioChek PA analyser Accu-chek guide me meter Accu-chek test strips x 200 Lipid panels x 15 40 μL collection tubes x 16 Glucose panels x 25 15 μL collection tubes x 25 UniStik 3 safety lancets x 100 Sharps safety bin 2 L Medical gloves medium x 100 Alcohol swabs x 200 Gauze swabs 5 cm × 5 cm (pack of 100) Band aids x 100 Portable stadiometer Gym rehabilitation equipment: Treadmill, exercise bikes, recumbent bike, arm ergometer, weights, resistance bands Participants used a step-counting app on their smartphone if they had one to monitor and record daily steps (physical activity). If no step-counting app was installed on their smart phone the physiotherapist helped install a freely downloadable app.
What procedures	Health screen (health questionnaires and point of care testing for metabolic syndrome) Participation in orthopaedic rehabilitation group, based on a cardiac rehabilitation model with 4 exercise stations: Treadmill, bike, upper limb strength; lower limb strength; and a 10 min education/information session. Rehabilitation supplemented by: goal setting physical activity, monitoring of physical activity and access to online learning program. Consistent with usual care, participants were eligible to receive individualised appointments with the physiotherapist to address joint-specific impairments and limitations.
Who provided	Physiotherapist with training in point of care testing by completing relevant modules on the Australian point of care Practitioner's network: https://www.appn.net.au/Physiotherapist and allied health assistant to lead exercise group. Allied health professionals including physiotherapist and dietitian provide educational component of the rehabilitation program on lifestyle issues such as physical activity, healthy diet, knee and hip health.
How provided	Face-to-face initial physiotherapy assessment (health screen) Face-to face-group exercise and education participation Face-to-face group education supplemented with online learning program. Physical activity goals setting and feedback provided using step counting app.
Where (setting)	Gymnasium in the community rehabilitation centre
When/how much (dose)	Initial joint assessment: Within 1 week of hospital discharge 1 to 2 physiotherapy usual care one-on-one sessions depending on progress Pre-group assessment (metabolic screen) prior to commencing group exercise group attendance: Twice weekly for 1 h for 6 weeks
Tailoring	Sessions tailored to the needs and progress of the individual including appropriate exercise at the 4 stations
Fidelity checking measures	Completeness of metabolic screen data; Number of completed sessions and content of sessions recorded in the medical record and scanned exercise sheet; Progress of self-monitored daily steps

The health screen was conducted by a physiotherapist who had completed training modules on the Australian Point of Care Practitioner's Network: https://www.appn.net.au/. It included measures of height, weight and waist circumference [25]; blood pressure [26]; triglycerides and HDL-C levels - capillary blood test using a CardioChek Analyser [27]; glucose – random blood glucose via capillary blood test using an Accu-chek my guide Analyser. If the participant had been diagnosed with diabetes or pre-diabetes or had a recent (within 3 months) fasting blood glucose test, the test was omitted and the participant was deemed to have this risk factor assessed. Similarly, the test was omitted if the participant had a recently documented blood test for triglycerides or cholesterol or were on medications for these conditions.

Group exercise duration was up to 50 min and consisted of two strengthening exercise stations and two aerobic exercise stations in line with cardiac rehabilitation models. Intensity of exercise was monitored with participants instructed to aim for a target heart rate of 50–70% of maximum heart rate or an exertion rating of 4–6 (i.e. moderate) on the 0–10 BORG perceived rating of exertion scale [28] (Table 1). Aerobic exercise increased in duration over the 12 sessions, for most starting at approximately 15 min increasing to more than 30 min by discharge. Strengthening exercises were progressed by increasing load. Based on the COM-B model of behaviour change [29], psychological capability to increase physical activity was addressed through attendance at brief 10 min education sessions at the end of one of the exercise group sessions each week. Participants received information on benefits of physical activity, increasing exercise safely, exercise guidelines, exercising with chronic conditions and a healthy diet. For diet, participants were encouraged to eat a healthy diet based on the Australian dietary guidelines [30]. For physical activity, participants were encouraged to meet physical activity guidelines of at least 150 min of moderate to vigorous intensity physical activity per week [2]. Participants were also provided access to electronic resources with more detail on these topics. Physical capability was addressed through supervised exercise rehabilitation; opportunity occurred through group exercise classes; and motivation provided through the physiotherapist acting as coach [21], setting goals related to both diet and physical activity and assisting achievement of physical activity recommendations through prescribing a home exercise program, which was monitored at group exercise sessions [29].

### 2.3 Outcomes

Outcomes were assessed at baseline (T0) and at the completion of the program (T1) by the physiotherapists who supervised the program. The primary outcome was feasibility, based on four domains of feasibility proposed by Bowen: acceptability, demand, implementation and practicality [22]. Bowen's framework was chosen as it provided guidance and structure to assess the viability and potential of PALMS in a pragmatic setting before a full-scale trial.

Acceptability refers to how those involved in PALMS perceived the program. Acceptability was measured via a survey for participants and healthcare professionals involved in the program at T1 [31]. The scale comprises eight items scored on a Likert scale from 1 (strongly disagree) to 5 (strongly agree) with higher scores indicating higher levels of acceptability.

Demand relates to the use of PALMS as an indicator of future demand for such a program. Demand was assessed as the percentage of all eligible clients referred to community rehabilitation in the study timeframe who participated in the program. The proportion of participants found to have metabolic syndrome at T0 was also calculated to determine the demand for a comprehensive lifestyle program following joint replacement surgery. Presence of metabolic syndrome was determined by the existence of three or more risk factors based on the joint international consensus statement [10] with triglyceride and blood glucose thresholds modified to suit random, non-fasting sampling techniques [32].

Implementation evaluated the degree to which the intervention was delivered as intended. It was recorded by measuring the proportion of scheduled sessions attended; and the proportion of participants who completed the program. Findings inform whether the program is feasible to be delivered as planned in the real-world setting or whether modifications need to occur before future iterations.

Practicality outcomes inform whether PALMS could be delivered safely and without impact to surrounding services. Practicality was measured by recording adverse events via the standard health service recording system and by clinician notes from each session. Adverse events were categorised as serious or non-serious, and related or not related to the intervention. Practicality was also assessed by monitoring the number of individual face-to-face appointments with the physiotherapist outside of the group program, and by comparing this with historical data for the same service.

Secondary outcomes comprised measures of physical activity, diet, knowledge and confidence about physical activity, measures of physical functioning and individual metabolic risk factors. Physical activity was measured using the self-administered International Physical Activity Questionnaire Short Form at T0 and T1 [33]. In addition, participants were asked to record daily steps from a wearable device or their smartphone, using the same method of measurement at both timepoints.

Diet was evaluated at T0 using the Commonwealth Scientific and Industrial Research Organisation Healthy Diet Score survey [34]. This online survey assesses individual's usual food intake consistent with Australian Dietary Guidelines across 38-items. A total diet score is estimated between 0 and 100, where a higher score reflects greater overall adherence to dietary guidelines.

The Self-Efficacy for Exercise Scale was used to assess participant's confidence in exercising at T0 and T1. This 9-item outcome is a self-reported, Likert rating scale that scores self-efficacy for exercise from 0 (not confident) to 10 (very confident). Higher scores correlate with higher levels of self-efficacy for exercise [35]. Minor wording modifications were made to ensure consistency with current physical activity recommendations.

Knowledge of physical activity was assessed at T0 and T1 using a self-reported multiple-choice questionnaire [36], measuring awareness and knowledge of current physical activity recommendations. Higher scores are associated with better knowledge of physical activity recommendations.

The Hip dysfunction and Osteoarthritis Outcome Score (HOOS-12) and Knee injury and Osteoarthritis Outcome Score (KOOS-12) are joint-replacement-relevant tools that provided a summary joint impact score [37] at T0 and T1. The minimal clinically important change ranges from 24.0 to 27.5 units/100 for the HOOS-12 and 17.5–21.9 units/100 for the KOOS-12 [38].

The 6-min walk test evaluated exercise capacity at T0 and T1. The minimal clinically important change after total knee joint replacement ranges from 74.3 to 88.6 m [39].

### 2.4 Statistical analysis

We aimed to recruit at least 30 participants, a sample size comparable to other feasibility trials, and sufficient for the primary aim of feasibility to be addressed [40].

The PALMS program was considered feasible if:

1. Participants and health care professionals found the program acceptable with a mean acceptability score ≥32/40 indicated by mean positive ratings (e.g. agree or strongly agree) across the 8 items
2. At least 50% of eligible clients participated in the study or a recruitment rate of at least 7 participants each month was achieved
3. At least 50% of eligible participants had metabolic syndrome, indicating demand for the program
4. The program was implemented as intended with participants completing on average at least two-thirds (8 of 12) of scheduled sessions; and that drop-outs or non-completions were fewer than 15% of the sample
5. The program was safe for participants recovering from hip or knee joint replacement surgery with no serious adverse events related to the intervention
6. The program did not result in an increase in individualised one-on one physiotherapy appointments required outside the program compared to the previous year.

Simple descriptive statistics were used to present sample characteristics, acceptability, demand, prevalence of metabolic syndrome, implementation and practicality. Change in secondary outcomes between baseline and follow-up were described as mean differences and 95% confidence intervals calculated in IBM SPSS version 28.0.

## 3 Results

**Fig. 1:**
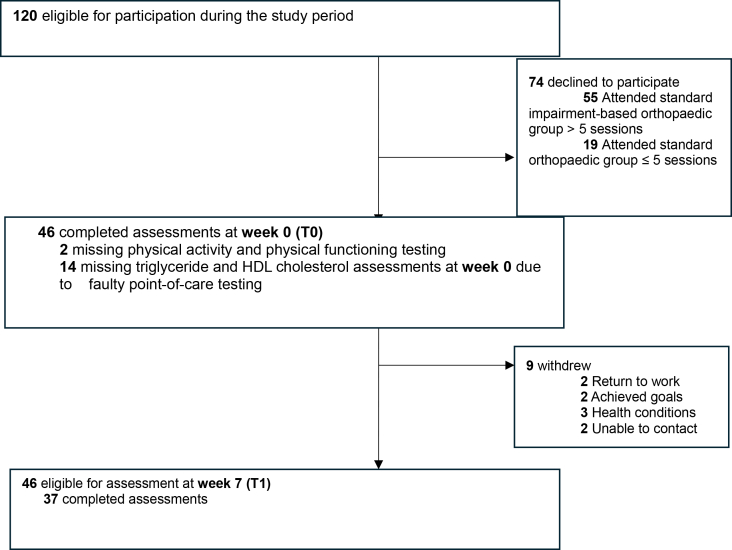
Flow of study participants through the study

Of the 120 eligible clients, 46 (23 men, 23 women, mean age of 62 [SD 8] years) recovering from total knee or hip joint replacement surgery participated in PALMS. The 74 eligible clients who did not participate in PALMS chose to participate in the standard impairment-focused orthopaedic group (Fig. 1). Participants commenced the program a mean of 7 (SD 4) weeks post total knee (n = 27) or hip (n = 19) joint replacement surgery (Table 2). At T0, 25 of 46 (55%) walked with a gait aid. During the program 9 participants (20%) withdrew and did not complete the T1 assessment. Reasons for withdrawal were health conditions (n = 3), returning to work (n = 2), achieving goals (n = 2) and not being able to be contacted (n = 2) (Fig. 1). On average, participants who withdrew were younger (mean difference 6 years, 95% CI 0.4 to 12) and were more likely to be walking without a gait aid at baseline, compared to participants who completed the T1 assessment (Table 2).

**Table 2:** Participant demographic characteristics at baseline.

Characteristic	Participants enrolled n = 46	Participants completed n = 37	Participants not completed n = 9	Comparison of completers and non-completers (Mean difference (95%CI) or Chi-square)
**Participants**				
**Age***(yr)*, mean (SD)	62 (8)	64 (8)	57 (8)	**6 (0.4, 12), *p*** = **0.038**
**Gender**, n males (%)	23 (50)	18 (49)	5 (56)	Χ^2^(1) = 0.138, *p* = 0.710
**Surgery**, n (%)				
TKR	27 (59)	23 (62)	4 (44)	Χ^2^(1) = 0.937, *p* = 0.333
Total hip joint replacement	19 (41)	14 (38)	5 (56)	
**Time post-surgery at baseline** (weeks), mean (SD)	7 (4)	6 (4)	8 (5)	−2 (−5, 1), *p* = 0.177
**Walking status** n (%)				
Walk unaided	21 (46)	14 (38)	7 (78)	**Χ^2^(1)** = **4.564, *p*** = **0.031**^a^
Walk with stick	3 (7)	3 (8)	0 (0)	
Walk with elbow crutches	19 (41)	18 (49)	1 (11)	
Walk with frame	3 (7)	2 (5)	1 (11)	
**PALMS sessions attended** (/12), mean (SD)	10.4 (3.0)	11.6 (1.1)	5.2 (3.2)	**6.4 (5.1, 7.6), p <0.001**
**1:1 physiotherapy sessions** mean (SD)	2.7 (2.6)	2.8 (2.8)	2.0 (1.5)	0.8 (−1.1, 2.8), *p* = 0.392

a Due to low observed cell frequencies compared walked unaided versus walked aided

At T0, 28 of the 37 (76%) participants with available data met the criteria for metabolic syndrome (Table 3). Nine participants had all 5 risk factors, 12 had 4 risk factors and 7 had 3 risk factors. Forty of 46 (87%) participants had hypertension, all (100%) had central obesity as measured by waist circumference and 11 of 46 (24%) had elevated blood glucose levels. Among participants with available lipid data from direct testing or chart review (n = 32), 78% had elevated triglycerides and 66% had low levels of HDL-C. A total of 18 of 40 (45%) diagnoses of hypertension were new. There were no statistical differences in proportion of completers and non-completers who had metabolic syndrome or metabolic risk factors (Table 3).

**Table 3:** Prevalence of metabolic syndrome and its risk factors at baseline.

Metabolic syndrome	Participants enrolled n = 46	Participants completed n = 37	Participants not completed n = 9	Comparison of completers and non-completers
**Risk factor**				
**Hypertension** n (%)	40 (87)	32 (86)	8 (89)	Χ^2^(1) = 0.037, *p* = 0.848
**Waist circumference** n (%)	46 (100)	37 (100)	9 (100)	Χ^2^(1) = 0.000, *p* = 1.000
**Glucose** n (%)	11 (24)	10 (27)	1 (11)	Χ^2^(1) = 1.008, *p* = 0.315
**Triglycerides** n (%)	Yes 25 (54) No 7 (15) Don't know 14 (30)	21 (57) 5 (14) 11 (30)	4 (44) 2 (22) 3 (33)	Χ^2^(1) = 0.567, *p* = 0.451^a^
**HDL cholesterol** n (%)	Yes 21 (46) No 11 (22) Don't know 14 (30)	17 (46) 9 (24) 11 (30)	4 (44) 2 (22) 3 (33)	Χ^2^(1) = 0.004, *p* = 0.953^a^
**Metabolic syndrome** n (%)				
Yes	28 (61)	24 (65)	4 (44)	Χ^2^(1) = 1.611, *p* = 0.204
No	9 (20)	6 (16)	3 (33)	
Don't know	9 (20)	7 (19)	2 (22)	

a Missing data for triglycerides and HDL Cholesterol due to point-of-care machine not functioning effectively; Criteria for presence of risk factors: abdominal obesity (defined by waist circumference values using current recommended thresholds for different populations) [10], elevated triglycerides (random serum triglyceride level ≥2.0 mmol/L or fasting levels ≥1.7 mmol/L, or taking medication for elevated triglycerides); reduced HDL cholesterol (HDL-C) (serum HDL-C < 1.0 mmol/L in males and <1.3 mmol/L in females, or taking medication for reduced HDL-C); elevated blood pressure (systolic ≥130 mmHg and/or diastolic ≥85 mmHg, or taking medication for hypertension); elevated blood glucose random ≥11.1 mmol/L [32], fasting ≥5.6 mmol/L, or diagnosed diabetes or pre-diabetes, or taking medication for elevated glucose).

### 3.1 Primary feasibility outcomes

Overall, the PALMS program was feasible with most feasibility threshold criteria fully met (Table 4).

**Table 4:** Primary outcome: Feasibility.

Feasibility domain	Outcome		Standard	Feasibility grading
**Acceptability**				
**Clients** (/40), mean (SD)	35.7 (3.6) n = 36		Clients and health professionals find the program acceptable	
**Clinicians***(/40),* mean (SD)	35.7 (1.4) n = 6			
**Demand**				
**Recruitment rate** n (%)	46 of 120 eligible participants (38%)		At least 50% of eligible participants participate	
**Recruitment rate***(/month)* mean (SD)[range]	5.1 (2.1) [range 2–9]		A recruitment rate of at least 7 participants each month is achieved	
**Metabolic syndrome***(presence of at least 3 risk factors)*, n (%)	28 of sample, 46 (61%) 28 of those tested, 36 (76%)		At least 50% participants have metabolic syndrome	
**Implementation**				
**Adherence** (*sessions attended/12*) mean (SD)	10.4 (3.0)		Clients completing on average at least two-thirds (8 of 12) of scheduled sessions	
**Drop-outs** n (%)	9 of 46 (19.6)		Drop-outs less than 15% of the sample	
**Practicality**				
**Adverse events** **Non-serious,** n (%) **Serious,** n (%)	**Related** 16 (35) 0 (0)	**Unrelated** 10 (17) 1 (2)	The program is safe for patients recovering from hip or knee joint replacement surgery	
**Other services (***1:1 physiotherapy consultations)* **PALMS** mean (SD**)** **Pre-PALMS** mean (SD)	2.7 (2.6), n = 46 2.4 (1.7), n = 25 Mean difference 0.3 (95%CI -0.9 to 1,4)		The program does not result in an increase in individualised one-on one physiotherapy appointments	

Feasibility threshold met ; Feasibility threshold partially met  Feasibility threshold not met

The program was reported to be highly *acceptable* by participants (n = 36), and clinicians (n = 6) who both recorded mean acceptability scores of 35.7/40.

There was *demand* for the program in that most (76%) participants had metabolic syndrome. Recruitment rates were not met as the 5.1 participants/month did not meet the specified threshold of 7; and only 38% of eligible clients agreed to participate. Despite this, the target sample was exceeded during the study period. The program was *implemented* with a high level of adherence with participants, on average, attending more than the threshold of 8 of the scheduled 12 sessions (mean 10.4/12). There were more dropouts than anticipated (9 of 46, 19.5%). However, 4 of the 9 who left the program before T1 were for positive reasons of having achieved their goals or returned to work. The program demonstrated *practicality* in that there were no serious adverse events related to the program. There were a small number of non-serious adverse events from more than 460 attendances, such as transient pain while exercising (n = 11), muscle pain (n = 2), swelling (n = 2), fatigue (n = 1) and low blood pressure (n = 1). There was no difference in the number of individual physiotherapy consultations between PALMS participants and a consecutive sample of clients attending the service one year previously (mean difference 0.3 consultations, 95%CI -0.8 to 1.4, *p* = 0.606) (Table 4).

### 3.2 Secondary outcomes

There were increases in physical activity from T0 to T1 (after completion of the program) in terms of self-reported METS min/wk (mean change 1,678, 95%CI 997 to 2,359) using the IPAQ-SF, and objectively measured daily steps (mean change 3,866, 95%CI 2,383 to 5,349) using wrist-worn devices or smartphone apps. The increases in levels of physical activity were accompanied by increases in self-efficacy and knowledge of physical activity (Table 5).

**Table 5:** Change in variables between admission and discharge from the program.

Characteristic	Admission T0 - baseline	Discharge T1 – week 7	Mean difference (95%CI)
**Metabolic risk factors**			
**Systolic blood pressure***(mmHg)*, mean (SD)	134.1 (18.1) n = 46	137.8 (16.0) n = 38	5.8 (−0.4, 11.9), *p* = 0.065
**Diastolic blood pressure***(mmHg)*, mean (SD)	79.9 (11.3) n = 46	85.2 (19.6) n = 38	**6.7 (0.1, 13.2), *p*** = **0.048**
**Glucose***(mmol/L),* mean (SD)	6.4 (2.3) n = 44	6.5 (1.9) n = 38	−0.4 (−1.1, 0.4), p = 0.336
**HDL cholesterol***(mmol/L)*, mean (SD)	1.31 (0.45) n = 22	–	-a
**Triglycerides** (*mmol/L),* mean (SD)	1.80 (0.84) n = 22	–	–
**Waist circumference***(cm),* mean (SD)	115.7 (15.7) n = 46	114.6 (15.0) n = 36	−0.8 (−1.8, 0.3), *p* = 0.158
**Body mass index***(kg/m*^*2*^*)***,** mean (SD)	35.3 (6.5) n = 46	34.9 (6.0) n = 36	**−0.2 (-0.4, -0.004) *p*** = **0.046**
**Physical activity**			
**Physical activity***(METS min/wk)***,** mean (SD)	1,451 (2,726) n = 44	2,690 (2,180) n = 37	**1,678 (997, 2,359), p <0.001**
**Physical activity level,** n (%)	High 4 (9) Moderate 21 (46) Low 19 (41) Unknown 2 (4)	High 14 (30) Moderate 18 (39) Low 5 (11) Unknown 9 (20)	**Χ^2^(1)** = **8.485, *p*** = **0.004**^b^
**Physical activity***(daily steps),* mean (SD)	2,013 (1304) n = 16	5,555 (2,452) n = 23	**3,866 (2,383, 5,349), p <0.001**
**Self-efficacy for exercise***(/90),* mean (SD)	42.5 (20.9) n = 44	54.5 (18.7) n = 37	**12.1 (4.0, 20.3), *p*** = **0.005**
**Knowledge of physical activity***(/12)*, mean (SD)	6.7 (1.9) n = 44	8.1 (1.7) n = 37	**1.5 (0.8, 2.2), p <0.001**
**Diet**			
**Healthy diet score,***(/100)*, mean (SD)	50.2 (11.8) n = 46	–	-c
**Physical function**			
**KOOS-12 summary score***(/100)* mean (SD)	48.3 (15.7) n = 26	69.3 (16.0) n = 23	**21.8 (12.5, 31.2), p <0.001**
**HOOS-12 summary score***(/100)*, mean (SD)	59.8 (18.8) n = 18	73.9 (19.6) n = 14	**17.6 (7.4, 27.7) *p*** = **0.002**
**6-min walk test,***(m)* mean (SD)	314 (109) n = 27	475 (97) n = 23	**167 (120, 215) p <0.001**

a Missing data for triglycerides and HDL Cholesterol due to point-of-care machine not functioning effectively;

b Physical activity distribution: low v moderate/high;

c Diet score only assessed on admission

Measures of physical function at the knee and hip also demonstrated likely clinically significant increases. The KOOS-12 summary score increased by 21.8 units (95%CI 12.5 to 31.2) and the HOOS-12 summary score increased by 17.4 units (95%CI 7.4 to 27.2). The 6-min walk test increased by a mean of 167 m (95%CI 120 to 215).

There was no consistent improvement in metabolic-associated risk factors over the 6-week program. There was a statistically significant reduction in body mass index (mean change −0.2 kg m^−2^, 95% CI -0.4 to −0.004) and statistically significant increase in diastolic blood pressure (mean change 6.7 mmHg, 95% CI 0.1 to 13.2). At T0 mean Commonwealth Scientific and Industrial Research Organisation diet score was 50.2 (SD 11.8) out of 100, indicating poor adherence to Australian dietary guidelines.

## 4 Discussion

The introduction of a twice-weekly, six-week physical activity and lifestyle management after joint replacement surgery group program in a publicly funded, community rehabilitation program was feasible. The program was well accepted by both participants and clinicians, was implemented with high rates of adherence, with a high demand in that up to three-quarters of participants had metabolic syndrome. However, recruitment rates only partially or did not meet threshold targets. Practicality of the program was demonstrated by the lack of serious adverse events and evidence that the program did not result in a shifting of resources to one-on-one consultations with a physiotherapist. Participation in the program was associated with likely clinically significant increases in physical activity and physical functioning; suggesting the change of rehabilitation focus from impairment to holistic did not compromise the trajectory of physical recovery after joint replacement surgery.

The high prevalence of metabolic syndrome highlights the need for rehabilitation programs that address broader metabolic health, rather than focusing on joint-specific impairments and activity limitations related to the replaced knee or hip. Program feasibility and the high levels of acceptance and engagement suggests that concerns that clients might only want to focus on their affected joint rather than their overall health may be ill-founded. The preliminary findings of increased levels of physical activity that were likely to be clinically significant [41] in a group where changes in physical activity have been resistant to change [3,4] are important; if maintained over a longer period these changes would be expected to mediate positive changes in metabolic risk factors [19]. Despite interest in multimorbidity rehabilitation as opposed to disease-specific rehabilitation across cardiovascular diseases [42] and some short-term benefits from incorporating motivational interviewing into conventional outpatient rehabilitation [43], to our knowledge this is the first study to evaluate applying a cardiac rehabilitation model of rehabilitation to patients recovering from joint replacement surgery.

Behaviour change elements in the PALMS program may have facilitated the observed increased levels of physical activity. Preliminary evidence is beginning to emerge that behaviour change interventions have the potential to increase physical activity levels after joint replacement [44,45]. These are likely important elements to consider in future programs.

We learnt valuable information to inform planning of a trial to evaluate effectiveness of the program on metabolic health. Recruitment rates at this single service suggest that for an adequately powered trial to be completed over 12 months, we would need to recruit from additional sites. Also, due to participant burden not all assessments were repeated at T1, which was scheduled immediately following the last group session. The timing of the final assessment immediately after the last exercise session might also explain the apparently aberrant finding of increased diastolic blood pressure. These issues could be addressed by adequately resourcing the assessment procedures with a dedicated assessor and time of assessment, and by choosing assessments necessary to address the trial question to avoid participant burden.

A strength of the study was that the feasibility criteria of the study were based on a framework [22] and had a priori benchmarks to evaluate feasibility. The study was conducted in a busy outpatient clinic, largely with existing resources. These pragmatic features increase generalisability and the likelihood that the program may be feasible in other health services. Although not part of this evaluation, eight months after the completion of the study the program, (including point-of-care glucose testing) continues to be offered with good uptake within the community rehabilitation program, suggesting that it has been incorporated into standard practice at this site. A limitation was the larger than expected amount of missing data on reassessment for some outcomes. The lack of follow up data on diet meant that this aspect of the program could not be evaluated. Related to this, point-of-care testing equipment for triglycerides and HDL-C was unreliable. However, other studies have demonstrated that point-of care testing can be implemented reliably [46] suggesting this is something that could be addressed in future studies. The absence of a control group makes it difficult to determine if changes observed are a result of the intervention or due to natural recovery following surgery. However, previous research suggests people return to their pre-surgical levels of physical activity by seven weeks with no further increases up to three months [47] or beyond [2,4].

In conclusion, a physical activity and lifestyle management after joint replacement surgery group program was feasible and associated with short-term improvements in physical activity and physical functioning. The next step is to conduct an adequately powered randomised controlled trial to evaluate medium term effectiveness in improving the health of people recovering from lower limb elective joint replacement surgery.

## Author contributions

All authors were involved in the conception and design of the study. Data collection and initial analyses were conducted by CB and NFT. NFT, DB and CLP wrote the first draft of the manuscript. All authors contributed to reviewing the manuscript and approved the submitted version.

## Data sharing statement

After final publication a de-identified data set will be supplied upon reasonable request to the corresponding author.

## Generative AI

Generative AI and AI-assisted technologies were NOT used in the preparation of this work.

## Role of funding sources

The study was partially supported by a small grant from the Victorian Department of Health, Australia (no application number).

## Declaration of competing interests

The authors have no competing interests to declare.

## References

[1] Nüesch E E., Dieppe P., Reichenbach S., Williams S., Iff S., Jüni P. (2011). All cause and disease specific mortality in patients with knee or hip osteoarthritis: population based cohort study. BMJ.

[2] Bull F.C., Al-Ansari S.S., Biddle S., Borodulin K., Buman M.P., Cardon G. (2020). World health organization guidelines on physical activity and sedentary behaviour. Br. J. Sports Med..

[3] Hammett T., Simonian A., Austin M., Butler R., Allen K.D., Ledbetter L L. (2018). Changes in physical activity after total hip or knee arthroplasty: a systematic review and meta-analysis of six- and twelve-month outcomes. Arthritis Care Res..

[4] Sašek M., Kozinc Z., Löfler S., Hofer C., Šarabon N. (2021). Objectively measured physical activity, sedentary behavior and functional performance before and after lower limb joint arthroplasty: a systematic review with meta-analysis. J. Clin. Med..

[5] Oatis C.A., Johnson J.K., DeWan T., Donahue K., Li W., Franklin P.D. (2019). Characteristics of usual physical therapy post-total knee replacement and their associations with functional outcomes. Arthritis Care Res..

[6] Jette D.U., Hunter S.J., Burkett L., Langham B., Logerstedt D.S., Piuzzi N.S. (2020). Physical therapist management of total knee arthroplasty. Phys. Ther..

[7] Grundy S.M., Hansen B., Smith S.C., Cleeman J.I., Kahn R.A. (2004). Clinical management of metabolic syndrome: report of the American heart association/national heart, lung, and blood institute/American diabetes association conference on scientific issues related to management. Arterioscler. Thromb. Vasc. Biol..

[8] Collins J.A., Diekman B.O., Loeser R.F. (2018). Targeting aging for disease modification in osteoarthritis. Curr. Opin. Rheumatol..

[9] Peiris C.L., Culvenor A.G. (2023). Metabolic syndrome and osteoarthritis: implications for the management of an increasingly common phenotype. Osteoarthr. Cartil..

[10] Alberti K.G., Eckel R.H., Grundy S.M., Zimmet P.Z., Cleeman J.I., Donato K.A. (2009). Harmonizing the metabolic syndrome: a joint interim statement of the International diabetes Federation task force on epidemiology and prevention; national heart, lung, and blood institute; American heart association; world heart Federation; International atherosclerosis society; and International association for the study of obesity. Circulation.

[11] Puenpatom R.A., Victor T.W. (2009). Increased prevalence of metabolic syndrome in individuals with osteoarthritis: an analysis of NHANES III data. Postgrad. Med. J..

[12] Shin D. (2014). Association between metabolic syndrome, radiographic knee osteoarthritis, and intensity of knee pain: results of a national survey. J. Clin. Endocrinol. Metab..

[13] Peiris C.L., Le V.A., Pazzinatto M.F., Heerey J., De Oliveira Silva D., Kemp J. (2025). Metabolic syndrome is associated with poorer outcomes in people with osteoarthritis participating in a rehabilitation program: an observational study. Disabil. Rehabil..

[14] Peiris C., Harding K., Porter J., Shields N., Gilfillan C., Taylor N. (2023). Understanding the hidden epidemic of metabolic syndrome in people accessing community rehabilitation: a cross-sectional study of physical activity, dietary intake, and health literacy. Disabil. Rehabil..

[15] Conley B., Bunzli S., Bullen J., O'Brien P., Persaud J., Gunatillake T. (2023). Core recommendations for osteoarthritis care: a systematic review of clinical practice guidelines. Arthritis Care Res..

[16] Gibbs A.J., Gray B., Wallis J.A., Taylor N.F., Kemp J.L., Hunter D.J. (2023). Recommendations for the management of hip and knee osteoarthritis: a systematic review of clinical practice guidelines. Osteoarthr. Cartil..

[17] Alhumaid A., Abdulaziz A., tayeb M., Alqhatani Y., Walbi I. (2025). Healthy lifestyle interventions for non-communicable disease prevention in SAudi Arabia: a scoping review. Cureus.

[18] Peiris C.L., van Namen M., O'Donoghue G. (2021). Education-based, lifestyle intervention programs with unsupervised exercise improve outcomes in adults with metabolic syndrome. A systematic review and meta-analysis. Rev. Endocr. Metab. Disord..

[19] van Namen M., Prendergast L., Peiris C. (2019). Supervised lifestyle intervention for people with metabolic syndrome improves outcomes and reduces individual risk factors of metabolic syndrome: a systematic review and meta-analysis. Metabolism.

[20] Eijsvogels T.M., Molossi S., Lee D.C., Emery M.S., Thompson P.D. (2016). Exercise at the extremes: the amount of exercise to reduce cardiovascular events. J. Am. Coll. Cardiol..

[21] Matthews J.A., Matthews S., Faries M.D., Wolever R.Q. (2024). Supporting sustainable health behavior change: the whole is greater than the sum of its parts. Mayo Clin Proc Innov Qual Outcomes.

[22] Bowen D.J., Kreuter M., Spring B., Cofta-Woerpel L., Linnan L., Weiner D D. (2009). How we design feasibility studies. Am. J. Prev. Med..

[23] von Elm E., Altman D.G., Egger M., Pocock S.J., Gøtzsche P.C., Vandenbroucke J.P. (2007). The strengthening the reporting of observational studies in epidemiology (STROBE) statement: guidelines for reporting observational studies. Ann. Intern. Med..

[24] Hoffmann T.C., Glasziou P.P., Boutron I., Milne R., Perera R., Moher D. (2014). Better reporting of interventions: template for intervention description and replication (TIDieR) checklist and guide. BMJ.

[25] Marfell-Jones M., Stewart A., de Ridder J. (2012). https://reviewbooku.com/review/international-standards-for-anthropometric-assessment-4951969.

[26] Stergiou G.S., Alpert B., Mieke S., Asmar R., Atkins N., Eckert S. (2018). A universal standard for the validation of blood pressure measuring devices: association for the advancement of medical instrumentation/European society of hypertension/International organization for standardization (AAMI/ESH/ISO) collaboration statement. Hypertension.

[27] dos Santos Ferreira C.E., França C.N., Correr C.J., Zucker M.L., Andriolo A., Scartezini M. (2015). Clinical correlation between a point-of-care testing system and laboratory automation for lipid profile. Clin. Chim. Acta.

[28] Williams N. (2017). The borg rating of perceived exertion (RPE) scale. Occup. Med..

[29] Michie S., van Stralen M.M., West R. (2011). The behaviour change wheel: a new method for characterising and designing behaviour change interventions. Implement. Sci..

[30] National Health & Medical Research Council (2013). https://www.eatforhealth.gov.au/guidelines/australian-dietary-guidelines-1-5.

[31] Sekhon M., Cartwright M., Francis J.J. (2022). Development of a theory-informed questionnaire to assess the acceptability of healthcare interventions. BMC Health Serv. Res..

[32] Moebus S., Hanisch J.U., Neuhäuser M., Aidelsburger P., Wasem J., Jöckel K.H. (2006). Assessing the prevalence of the metabolic syndrome according to NCEP ATP III in Germany: feasibility and quality aspects of a two step approach in 1550 randomly selected primary health care practices. Ger. Med. Sci..

[33] Craig C.L., Marshall A.L., Sjöström M., Bauman A.E., Booth M.L., Ainsworth B.E. (2003). International physical activity questionnaire: 12-country reliability and validity. Med. Sci. Sports Exerc..

[34] Hendrie G.A., Rebuli M.A., Golley R.K. (2017). Reliability and relative validity of a diet index score for adults derived from a self-reported short food survey. Nutr. Diet..

[35] Resnick B., Jenkins L.S. (2000). Testing the reliability and validity of the Self-Efficacy for Exercise scale. Nurs. Res..

[36] Deans S., Kirk A., McGarry A., Rowe D. (2020). Physical activity guidelines and promotion: an online survey of United Kingdom's prosthetic rehabilitation healthcare professionals. Prosthet. Orthot. Int..

[37] Ackerman I., Soh S., Harris I., Cashman K., Heath E., Lorimer M. (2021). Performance of the HOOS-12 and KOOS-12 instruments for evaluating outcomes from joint replacement surgery. Osteoarthr. Cartil..

[38] Soh S.E., Harris I.A., Cashman K., Heath E., Lorimer M., Graves S.E. (2022). Minimal clinically important changes in HOOS-12 and KOOS-12 scores following joint replacement. J Bone Joint Surg Am.

[39] King L.K., Hawker G.A., Stanaitis I., Woodhouse L., Jones C.A., Waugh E.J. (2022). Minimal clinically important difference for improvement in six-minute walk test in persons with knee osteoarthritis after total knee arthroplasty. BMC Muscoskelet. Disord..

[40] Lancaster G.A., Dodd S., Williamson P.R. (2004). Design and analysis of pilot studies: recommendations for good practice. J. Eval. Clin. Pract..

[41] White D.K., Tudor-Locke C., Zhang Y., Fielding R., LaValley M., Felson D.T. (2014). Daily walking and the risk of incident functional limitation in knee osteoarthritis: an observational study. Arthritis Care Res..

[42] Barker K., Holland A.E., Lee A.L., Haines T., Ritchie K., Boote C. (2018). Multimorbidity rehabilitation versus disease-specific rehabilitation in people with chronic diseases: a pilot randomized controlled trial. Pilot Feasibility Stud..

[43] Christiansen C.L., Kline P.W., Anderson C.B., Melanson E.L., Sullivan W.J., Richardson V.L. (2024). Optimizing total knee arthroplasty rehabilitation with telehealth physical activity behavior change intervention: a randomized clinical trial. Phys. Ther..

[44] Bird M.L., Mulford J., Williams A.D., Cheney M., O'Brien J. (2023). Adding behaviour-change counselling to an exercise program for adults preparing for hip and knee arthroplasty improves psychological and physical wellness: focus group reflections. Int. J. Environ. Res. Publ. Health.

[45] Hawke L.J., Shields N., Dowsey M.M., Choong P.F.M., Taylor N.F. (2019). Physical activity levels after hip and knee joint replacement surgery: an observational study. Clin. Rheumatol..

[46] Mastwyk S.E., Taylor N.F., Lowe A., Dalton C.F., Peiris C.L. (2025). Metabolic health screening in physical therapy private practice in Australia: a feasibility study. Phys. Ther..

[47] Lebleu J., Poilvache H., Mahaudens P., De Ridder R., Detrembleur C. (2021). Predicting physical activity recovery after hip and knee arthroplasty? A longitudinal cohort study. Braz. J. Phys. Ther..

